# Eucalyptus Trees in the Roman Campagna

**Published:** 1883-03

**Authors:** 


### EUCALYPTUS TREES IN THE ROMAN CAMPAGNA.

An interesting debate took place in the Italian Parliament last week on the employment of convicts in agricultural operations, which has been going on for some years at the Abbey of the Tre Fontaine in the Roman Campagna, under the direction of the Trappists. The object with which these works were started was to ascertain if the cultivation of the soil would result in purifying the air of the Campagna, the general opinion being that the malaria is caused by atmospheric influences unsusceptible of modification. The results so far have been most satisfactory. At first the monks were obliged to live within the city walls during the bad season; but since the ground has come under cultivation, and, above all, since the eucalyptus globulus has been planted on a large scale in the neighborhood, the Abbey has been inhabited all the year round, and the fevers from which its inmates still sometimes suffer are of a mild character, and rarely fatal; whereas, at the outset, something like a fourth of the little community succumbed every year. The debate proved that the health of the two hundred and eighty convicts employed on the works was satisfactory, the average annual deaths from malaria not exceeding three. The government has lately made a very large grant of land in perpetuity to the Trappists, who have already planted on it no fewer than 100,000 eucalyptus trees, which are all doing well.

